# Hypogalactia after delivery: A man-made problem

**DOI:** 10.7189/jogh.13.03053

**Published:** 2023-11-09

**Authors:** Sophie Jullien, Irina Mateescu, Susanne Carai

**Affiliations:** 1 World Health Organization Regional Office for Europe, Quality of care and patient safety office, Athens, Greece; 2 World Health Organization Regional Office for Europe, Child and Adolescent Health, Copenhagen, Denmark; 3 World Health Organization Romania country office, Bucharest, Romania; 4 Witten Herdecke University, Witten, Germany

**Figure:**
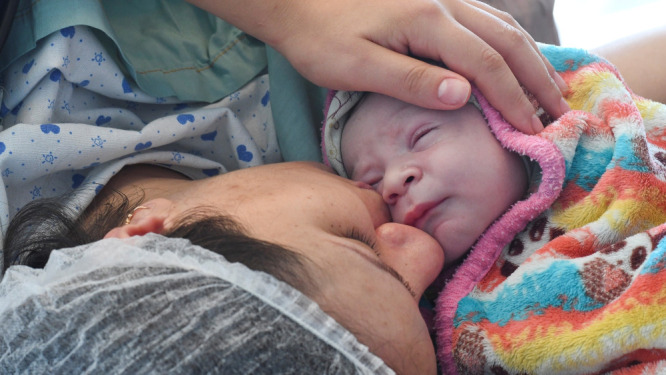
Photo: In Romania, babies are often routinely separated from their mothers after birth, contributing to hypogalactia. Source: World Health Organization, by Malin Bring. Used with permission.

Breastfeeding has incontestable proven health benefits for mothers and babies [1], yet less than half of all babies globally benefit from optimal breastfeeding [2]. The recent Lancet Series on breastfeeding examines social, political, ideological, and economic reasons underlying this phenomenon [3]. Poor healthcare, including the medicalisation of birthing and infant care, is reported as one of the key structural barriers that undermine the breastfeeding environment. Based on our recent health system evaluation conducted across 10 public hospitals in Romania in December 2021 and the ensuing review of 240 randomly selected medical records, 44.2% of women who gave birth were found to be discharged with a diagnosis of hypogalactia, defined as insufficient milk secretion to maintain exclusive breastfeeding [4]. During this evaluation, observations of clinical practices in the delivery rooms and obstetric wards and conversations with health workers revealed poor healthcare as a key barrier to breastfeeding. In all hospitals, newborns were separated from their mothers just after birth, for at least one hour after vaginal delivery (at least two hours in most hospitals visited) and at least 12 hours after caesarean section. The initiation of breastfeeding was almost invariably delayed, and overall, there was a lack of breastfeeding support for mothers from health staff. These practices are not in line with international standards of care and are likely the main reasons for the high proportion of women labeled as having insufficient milk to exclusively breastfeed their baby (hypogalactia). The World Health Organization (WHO) and other international bodies strongly recommend keeping babies in skin-to-skin contact with their mothers during the first hour after birth to promote breastfeeding, and to put all babies to the breast as soon as possible, unless clinically unstable [5,6].

Most mothers and babies do not need medical treatment, but do require professional support during the first hours of the baby’s life. The absence or suboptimal provision of such support potentially leads to avoidable harm with crucial consequences, such as mothers who cannot feed their own babies.

The Lancet breastfeeding Series likewise states parents and health professionals frequently misinterpret normal baby behaviours, such as crying and short night-time sleep durations, as signs of milk insufficiency or inadequacy [3]. Researchers in the Romanian study could not differentiate whether the diagnosis of hypogalactia reported in the medical records was based on a diagnosis made by a health professional or based on self-reported information of insufficient milk from the mother [4].

Actions are needed for improving the education of health workers and families on breastfeeding practices and beliefs, and toward the adoption and effective implementation of evidence-based recommendations for improving quality of care for mothers and newborns in Romania. This will require changing practices and behaviours. Addressing the shortage of midwives and their undervalued role is key in this task [7]. Indeed, all these breastfeeding supportive practices are within the remit of midwifery competencies. There is evidence of the impact of well-trained midwives in improving quality of care and in reducing maternal and newborn deaths [8]. Yet Romania has only 1.6 midwives per 10 000 population, much below the WHO regional average of 4.1 [9]. Countries should recognise the essential role of well-trained midwives for maternal, newborn, sexual, and reproductive health and define their roles and responsibilities within an interprofessional team. This will contribute to preventing medicalisation of practice such as we have observed in Romania, and ensure evidence-based essential care of healthy mothers and their babies, including adequate support for breastfeeding [8,10].
